# When do we need to suspect maturity onset diabetes of the young in patients with type 2 diabetes mellitus?

**DOI:** 10.20945/2359-3997000000468

**Published:** 2022-04-28

Arch Endocrinol Metab. 2022;66(1):32-9

Where you read:

**Özlem Üstay** ^1^

https://orcid.org/0000-0002-7993-955X

**Tugçe Apaydin** ^1^

https://orcid.org/0000-0001-9277-8669

**Onur Elbasan** ^1^

https://orcid.org/0000-0001-8580-9471

**Hamza Polat** ^2^

https://orcid.org/0000-0003-0833-9968

**Gizem Günhan** ^3^

https://orcid.org/0000-0001-6592-7171

**Ceyda Dinçer** ^1^

https://orcid.org/0000-0003-3680-3051

**Lamia Şeker** ^3^

https://orcid.org/0000-0003-3838-1753

**Esra Arslan Ateş** ^2^

https://orcid.org/0000-0001-5552-8134

**Ayşegül Yabacı** ^4^

https://orcid.org/0000-0002-5813-3397

**Ahmet Ilter Güney** ^2^

https://orcid.org/0000-0002-1661-1282

**Dilek Gogas Yavuz** ^1^

https://orcid.org/0000-0002-0075-6313

Should read:

**Özlem Üstay** ^1^

https://orcid.org/0000-0002-7993-955X

**Esra Arslan Ateş** ^2^

https://orcid.org/0000-0001-5552-8134

**Tugçe Apaydin** ^1^

https://orcid.org/0000-0001-9277-8669

**Onur Elbasan** ^1^

https://orcid.org/0000-0001-8580-9471

**Hamza Polat** ^2^

https://orcid.org/0000-0003-0833-9968

**Gizem Günhan** ^3^

https://orcid.org/0000-0001-6592-7171

**Ceyda Dinçer** ^1^

https://orcid.org/0000-0003-3680-3051

**Lamia Şeker** ^3^

https://orcid.org/0000-0003-3838-1753

**Ayşegül Yabacı** ^4^

https://orcid.org/0000-0002-5813-3397

**Ahmet Ilter Güney** ^2^

https://orcid.org/0000-0002-1661-1282

**Dilek Gogas Yavuz** ^1^

https://orcid.org/0000-0002-0075-6313

